# Ablation of Death-Associated Protein Kinase 1 Changes the Transcriptomic Profile and Alters Neural-Related Pathways in the Brain

**DOI:** 10.3390/ijms24076542

**Published:** 2023-03-31

**Authors:** Ruomeng Li, Shuai Zhi, Guihua Lan, Xiaotong Chen, Xiuzhi Zheng, Li Hu, Long Wang, Tao Zhang, Tae Ho Lee, Shitao Rao, Dongmei Chen

**Affiliations:** 1 Fujian Key Laboratory of Translational Research in Cancer and Neurodegenerative Diseases, Institute of Basic Medicine, School of Basic Medical Sciences, Fujian Medical University, Fuzhou 350122, Chinatlee0813@fjmu.edu.cn (T.H.L.); 2 Department of Bioinformatics, Fujian Key Laboratory of Medical Bioinformatics, School of Medical Technology and Engineering, Fujian Medical University, Fuzhou 350122, China; 3 Key Laboratory of Ministry of Education for Gastrointestinal Cancer, School of Basic Medical Sciences, Fujian Medical University, Fuzhou 350122, China

**Keywords:** death-associated protein kinase 1 (DAPK1), differential transcriptional profiling, brain, neurodegeneration, neuronal functions

## Abstract

Death-associated protein kinase 1 (DAPK1), a Ca^2+^/calmodulin-dependent serine/threonine kinase, mediates various neuronal functions, including cell death. Abnormal upregulation of DAPK1 is observed in human patients with neurological diseases, such as Alzheimer’s disease (AD) and epilepsy. Ablation of DAPK1 expression and suppression of DAPK1 activity attenuates neuropathology and behavior impairments. However, whether DAPK1 regulates gene expression in the brain, and whether its gene profile is implicated in neuronal disorders, remains elusive. To reveal the function and pathogenic role of DAPK1 in neurological diseases in the brain, differential transcriptional profiling was performed in the brains of DAPK1 knockout (DAPK1-KO) mice compared with those of wild-type (WT) mice by RNA sequencing. We showed significantly altered genes in the cerebral cortex, hippocampus, brain stem, and cerebellum of both male and female DAPK1-KO mice compared to those in WT mice, respectively. The genes are implicated in multiple neural-related pathways, including: AD, Parkinson’s disease (PD), Huntington’s disease (HD), neurodegeneration, glutamatergic synapse, and GABAergic synapse pathways. Moreover, our findings imply that the potassium voltage-gated channel subfamily A member 1 (Kcna1) may be involved in the modulation of DAPK1 in epilepsy. Our study provides insight into the pathological role of DAPK1 in the regulatory networks in the brain and new therapeutic strategies for the treatment of neurological diseases.

## 1. Introduction

Death-associated protein kinase 1 (DAPK1) belonging to the DAPK family, is a calcium/calmodulin-regulated serine/threonine protein kinase that is encoded by the *DAP* gene located on chromosome 5 and was originally discovered and identified as necessary for interferon gamma (IFNγ)-mediated apoptosis in HeLa cells [1,2,3]. DAPK1 is a 160 kDa multidomain enzyme that is composed of a kinase domain, a calcium/calmodulin binding domain, ankyrin repeats, putative P-loops, a Ras of complex (ROC) domain, a C-terminal of ROC (COR) domain, a death domain, and a serine-rich C-terminal tail [4]. DAPK1 is widely expressed in almost all tissues of mice and rats, and it is especially abundant in the adult and developing embryonic brain as well as the lung [5]. Substantial evidence indicates that DAPK1 is involved in apoptosis, autophagy, necrosis, and anoikis-like cell death [4,6,7,8]. Both DAPK1 knockout (DAPK1-KO) and the inhibition of DAPK1 function protects neurons against neuronal damage, whereas DAPK1 overexpression induces cell death [9,10,11,12]. In addition, DAPK1 may be involved in neurogenesis and other neuronal functions, such as synaptic transmission and plasticity, as well as cognition [5,6,13,14,15,16].

A large number of studies have proven that DAPK1 plays an Important role in mediating the pathological process of acute and chronic neurological disorders, such as Alzheimer’s disease (AD) [9,17,18,19,20], Parkinson’s disease (PD) [21,22], Huntington’s disease (HD) [23], traumatic brain injury (TBI) [24,25], stroke [15,26,27,28], and epilepsy [10,11]. We discovered that levels of DAPK1 are significantly increased in the hippocampi of 75% of AD patients compared to those in control subject samples [17,19,20]. Activation of DAPK1 results in Aβ pathology characteristic of AD through the amyloidogenic processing of APP, hyperphosphorylation and dysregulation of tau, and cell death by multiple pathways in AD, while DAPK1 inhibition is able to attenuate AD-related pathologies [8,17,19,20,29,30,31]. Moreover, activation of DAPK1 contributes to learning and memory deficiency, whereas inhibition of DAPK1 through deletion of the DAPK1 kinase domain ameliorates learning and memory impairment in mice [16,32,33]. DAPK1 has also been shown to be involved in interleukin (IL)-1β release, which plays a critical role in inflammatory and immune responses in the central nervous system (CNS) by regulating inflammasome activation in microglial cells and in mice treated and injected with Aβ_25–35_, respectively [34]. However, DAPK1 knockdown and catalytic activity inhibition decrease inflammation and ameliorate memory impairment [34]. In addition, DAPK1 upregulation promotes dopaminergic neuron loss and oxidative stress in PD [21,35]. In contrast, inhibition of DAPK1 reverses the impact on neuronal loss [22,35]. Similar to AD and PD, a reduction in DAPK1 activity prevents the loss of dendritic spines and the synapse dysfunction by normalizing the phosphorylation of extrasynaptic N-methyl-D-aspartate (NMDA) receptors in HD [23]. It has also been reported that DAPK1 promotes neuropathology after TBI, whereas suppression of both DAPK1 expression and kinase activity significantly attenuates neuronal apoptosis, synaptic loss, and cognitive impairments in TBI model mice [24,25]. In addition, DAPK1 modulates brain damage via the NMDA receptor or alpha-amino-3-hydroxy-5-methyl-4-isoxazole propionic acid (AMPA) receptor by ischemia and inhibition of DAPK1 activity, which contributes to neuroprotective effects in a mouse model of ischemia [15,28,36,37]. Furthermore, DAPK1 expression is dramatically elevated in brain samples of epileptic patients compared with age-matched healthy individuals [38,39]. Previous reports have shown that DAPK1 promotes neuronal apoptosis in seizures by interacting with tumor necrosis factor receptor 1 (TNFR1) or p53 [40,41]. We have recently reported that depletion of DAPK1 expression and inhibition of DAPK1 activity dramatically reduces epileptic seizures in mice after convulsive pentylenetetrazol or glutamate analog kainic acid exposure [10,11]. In summary, DAPK1 upregulation promotes neuronal damage, whereas downregulation of DAPK1 is beneficial for neuronal functions, which suggests that DAPK1 inhibition might exert therapeutic effects on many neurological diseases. However, the effects of DAPK1 regulation on the functions of global genes are still unclear.

In the present study, we report for the first time the function of DAPK1 in the brain and the role of DAPK1 in neurological diseases through transcriptional profiling of DAPK1-KO mice. Global gene expression was examined in different brain regions of DAPK1-KO and wild-type (WT) mice and the differentially expressed genes (DEGs) were analyzed by Gene Ontology (GO) enrichment and Kyoto Encyclopedia of Genes and Genomes (KEGG) pathway enrichment analyses. The results not only showed that DAPK1 plays a role in synaptic, cognitive and neuronal death-associated neuronal functions but also showed that DAPK1 is implicated in neurological diseases. Thus, our study demonstrates that the DAPK1 may contribute to the pathogenesis of neurological diseases and could be a potential target for neurological diseases therapies.

## 2. Results

### 2.1. Transcriptional Profiling in DAPK1-KO Mice

**Figure 1:**
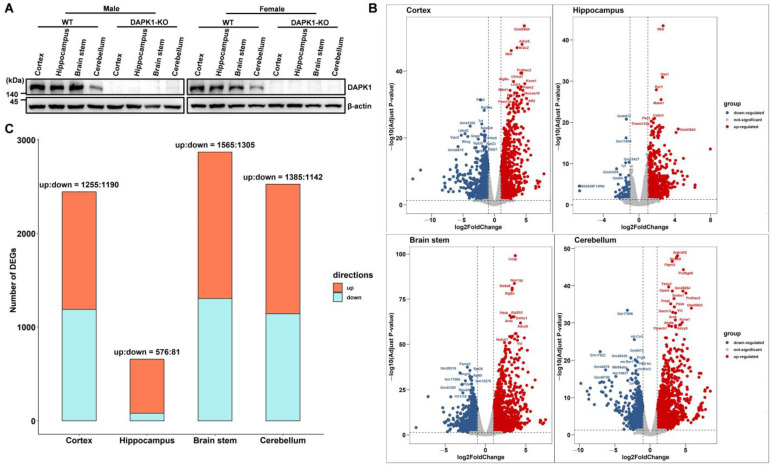
Transcriptional profiling of tissues from different brain regions in DAPK1-KO mice. (**A**) DAPK1 protein levels in tissues from four different brain regions of WT and DAPK1-KO male and female mice by immunoblotting analysis. (**B**) Differential gene expression volcano plots of tissues from each brain region of male mice by the edgeR method. The horizontal dotted line refers to the threshold of statistical significance with log, while the vertical dotted line refers to the threshold of the differential expressed ratio. (**C**) Number of DEGs in tissues of four brain regions for male mice.

To investigate the role of DAPK1 in global gene expression changes, RNA sequencing (RNA-seq) was performed in different brain tissues, including the cerebral cortex, hippocampus, brain stem, and cerebellum, in male and female DAPK1-KO and WT mice. DAPK1 expression was distributed in all four brain regions in WT mice, although the expression level of DAPK1 in the cerebellum was lower than that in the other brain regions for both males and females. However, DAPK1 was not expressed in the brain tissues of either male or female DAPK1-KO mice (Figure 1A). DEGs analyzed by the R software ‘limma’ package (v3.28.14) and ‘edgeR’ package (v3.14.0) are shown in Supplemental File S1. Moreover, DEGs by overlapping these two methods are shown in Supplemental File S2. The analysis of DEGs by a volcano plot revealed that 2445, 657, 2870, and 2527 genes were significantly altered in the cerebral cortex, hippocampus, brain stem, and cerebellum of male DAPK1-KO mice, respectively (Figure 1B for edgeR and Figure S1A for limma). Moreover, 1940, 881, 1931, and 2539 genes were significantly altered in the cerebral cortex, hippocampus, brain stem, and cerebellum of female DAPK1-KO mice, respectively. (Figure S2). In addition, the number of regulated genes, including upregulated genes and downregulated genes, for each type of brain tissue is shown in Figure 1C and Figure S1B for males and females, respectively. As the data showed, the number of upregulated genes was more than that of downregulated genes, especially in the hippocampus. Interestingly, the hippocampus showed the lowest number of DEGs for both males and females (Figure 1C and Figure S1B). Moreover, the fold-change was lower in the hippocampus than in the other brain regions. 

**Figure 2:**
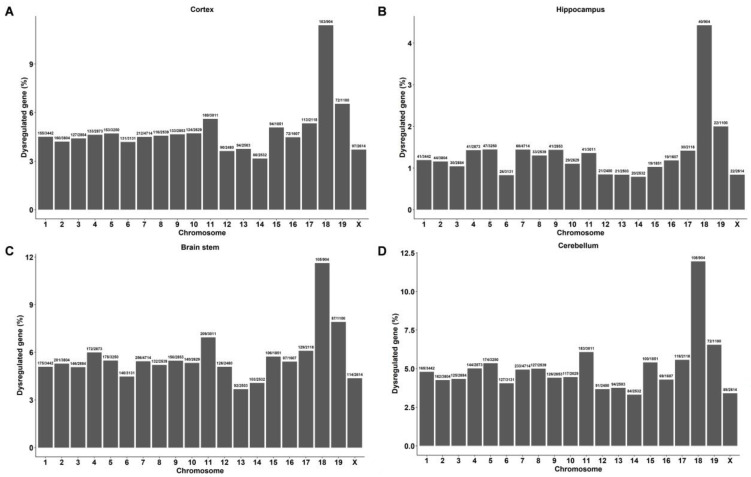
Chromosome distribution of significantly regulated genes in different regions of male DAPK1-KO mouse brain tissues. Chromosome distribution of DEGs in the cerebral cortex (**A**), hippocampus (**B**), brain stem (**C**) and cerebellum (**D**).

We further analyzed the number of DEGs in each chromosome and found that the DEGs in the brain tissues of DAPK1-KO mice were distributed on 20 chromosomes, including 19 autosomes and 1 sex chromosome (Figure 2 and Figure S3). The data showed that the number of DEGs on chromosome 7 was the highest in all brain tissues for both males and females; however, the highest percentage of DEGs existed on chromosome 18. Moreover, the common DEGs among all four brain regions were also distributed on 20 chromosomes, and chromosome 18 showed both the highest number and the highest percentage of common DEGs (Figure S4). Furthermore, DEGs were not only distributed on 20 chromosomes but also in the mitochondria. 

### 2.2. Common and Unique DEGs in the Tissues from Four Brain Regions

**Figure 3:**
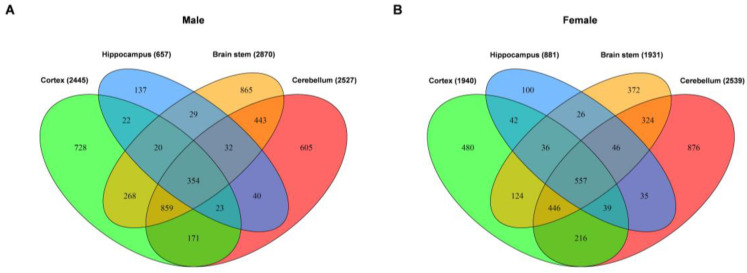
Venn analysis of the differentially expressed genes in different brain region tissues of male (**A**) and female (**B**) DAPK1-KO mice.

To further identify the common and unique DEGs in the tissues from four brain regions, we analyzed the DEGs by Venn diagram. The overlap of all DEGs for males and females is shown in Figure 3A,B. We found that most of the DEGs were unique to the cerebellum. Moreover, the figure shows that 354 and 557 DEGs were common in the tissues from all four brain regions for males and females, respectively. Moreover, 934 and 567 genes were significantly dysregulated in the tissues of at least three brain regions for males and females, respectively. Interestingly, the cerebral cortex, brain stem and cerebellum had the highest number of common DEGs when comparing the number of shared DEGs within all four brain regions. Furthermore, the hippocampus tissue showed the lowest number of DEGs (657 genes for males and 881 genes for females), almost all of which (~54% for males and ~63% for females) were shared with the tissues from the other brain regions. Detail information of Venn diagram is shown in Supplemental Files S3 and S4 for males and females, respectively.

### 2.3. GO Enrichment Analysis of DEGs

To explore the potential function of DEGs in the brains of DAPK1-KO mice, GO enrichment analysis was performed on the tissues of the four brain regions by analyzing them according to three categories: biological process, cellular component, and molecular function. When comparing DAPK1-KO and WT mice, there were 983 (727 for biological processes, 139 for cell component, and 117 for molecular function) and 244 (156 for biological processes, 61 for cell component, and 27 for molecular function) GO categories that were enriched in the DEGs in the cerebral cortex for males and females, respectively. The number of enriched GO categories in other brain tissues is listed in Table 1. The data indicated that DAPK1-KO has a larger impact on male mice than on female mice, and that DAPK1-KO affects gene regulation in the hippocampus less than other brain regions.

**Figure 4:**
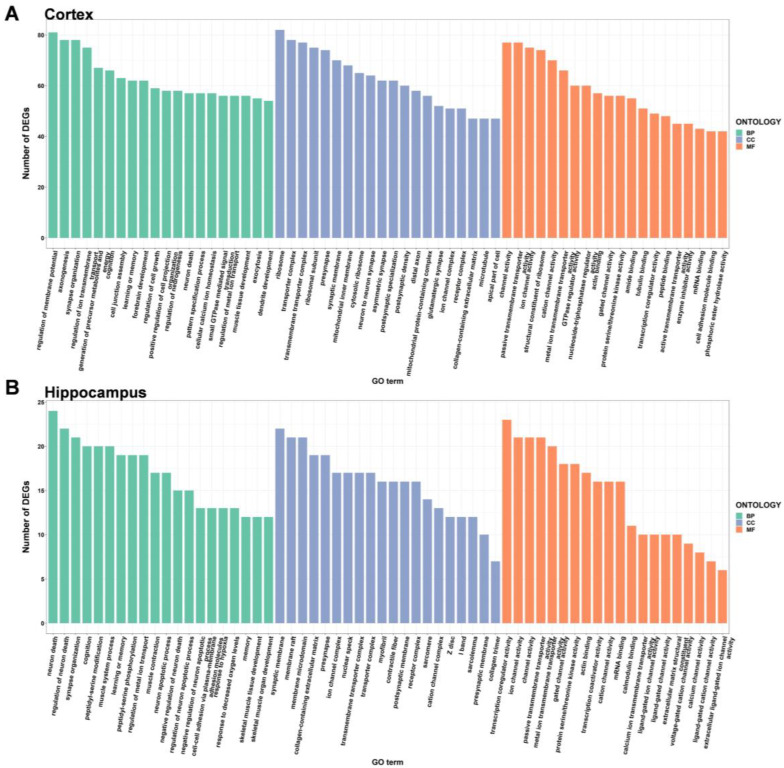
Gene ontology enrichment analysis of the DEGs in the cerebral cortex (**A**) and hippocampus (**B**) of male DAPK1-KO mice. The GO categories were biological process (BP), cellular component (CC), and molecular function (MF).

**Figure 5:**
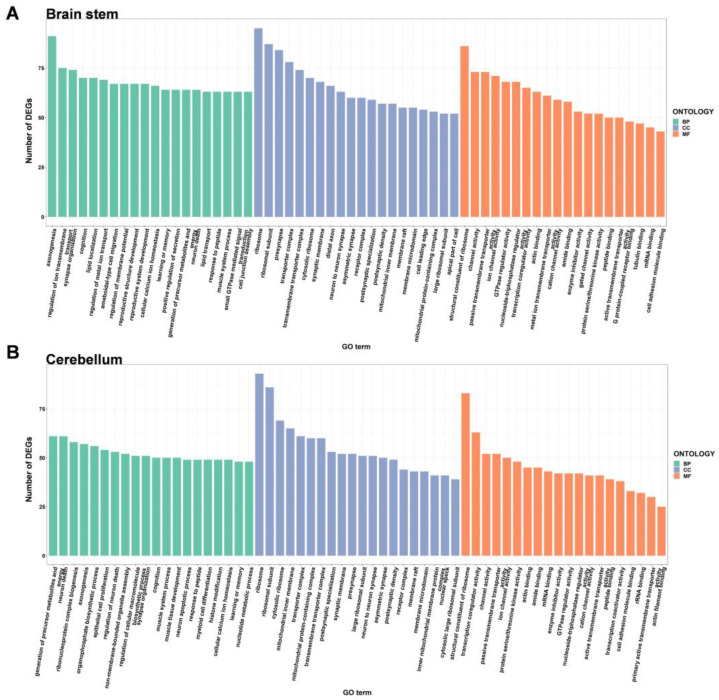
Gene ontology enrichment analysis of the DEGs in the brain stem (**A**) and cerebellum (**B**) of male DAPK1-KO mice. The GO categories were biological process (BP), cellular component (CC), and molecular function (MF).

GO enrichment analysis depicted the top 20 GO terms for each type depending on the number of DEGs in males and females (Figure 4, Figure 5, Figures S5 and S6). Compared to WT mice, the top three biological process terms significantly changed in the cerebral cortex of DAPK1-KO mice were regulation of membrane potential, learning or memory, and cognition. The top three cellular component terms significantly changed were cytosolic ribosome, ribosomal subunit, and ribosome, while structural constituent of ribosome, cation channel activity, and ion channel activity were the top three significantly changed molecular function terms. For the hippocampus, the top three significantly changed biological process terms included neuron death, regulation of neuron death, and learning or memory, and the top three enriched terms in cellular component were synaptic membrane, myofibril, and Z disc, while transcription coactivator activity, transcription coregulator activity, and gated channel activity were the top three molecular function terms. For the brain stem, axonogenesis, cognition, and learning or memory were the top three significantly enriched biological process terms, while the top three cellular component and molecular function terms were the same as those in the cerebral cortex. For the cerebellum, generation of precursor metabolites and energy, ribonucleoprotein complex biogenesis, and neuron death were the top three biological process terms significantly enriched. The top three molecular function terms were structural constituent of ribosome, rRNA binding, and primary active transmembrane transporter activity, while the top three cellular component terms were the same as those in the cerebral cortex and brain stem. Detailed information of GO enrichment analysis is shown in Supplemental File S5.

### 2.4. KEGG Pathway Analysis of DEGs

**Figure 6:**
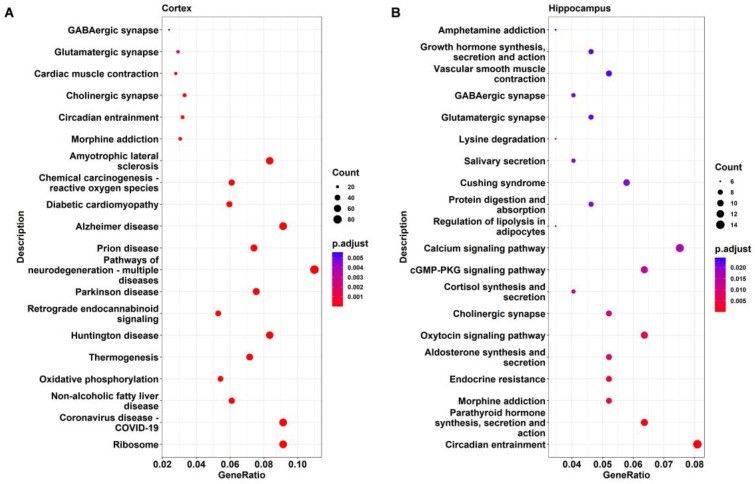
KEGG pathway analysis of the DEGs in the cerebral cortex (**A**) and hippocampus (**B**) of male DAPK1-KO mice.

**Figure 7:**
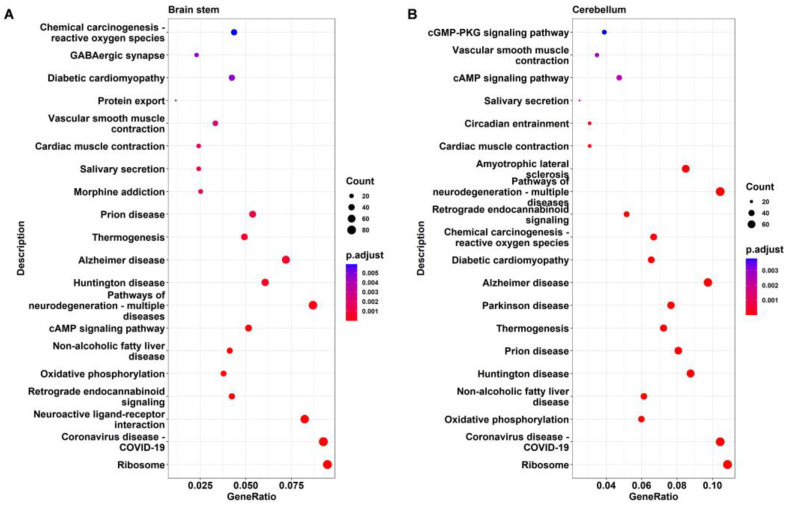
KEGG pathway analysis of the DEGs in the brain stem (**A**) and cerebellum (**B**) of male DAPK1-KO mice.

KEGG analysis was applied to further identify the important pathways associated with the DEGs. The top 20 significantly enriched pathways in DAPK1-KO mice compared with WT mice for males and females are depicted in Figure 6, Figure 7, Figures S7 and S8, respectively. The KEGG analysis revealed that several pathways, including ribosome-related, coronavirus disease 2019 (COVID-19), age-associated, nonalcoholic fatty liver disease (NAFLD), and oxidative phosphorylation pathways were significantly enriched in the cerebral cortex, brain stem, and cerebellum, but not in the hippocampus. Similarly affected were many neurodegenerative disease-related pathways, such as AD, PD, and HD. COVID-19 was the most significantly enriched KEGG pathway in the cerebral cortex, brain stem, and cerebellum, with 69, 81, and 75 related genes for DAPK1-KO males (49, 52, and 75 DEGs for DAPK1-KO females), respectively. Moreover, the main enriched pathways in the hippocampus were endocrine-related pathways, such as morphine addiction, salivary secretion, and parathyroid hormone synthesis, secretion, and action. We also found that circadian entrainment and synapse-related pathways, including glutamatergic synapses and GABAergic synapses, were significantly enriched in almost all tissues. Consistent with the number of DEGs and GO terms, the number of significant KEGG pathways was smaller in the hippocampus than those in other brain regions, especially the female hippocampus. However, the types of KEGG pathways enriched in males and females were similar in each brain region. Detailed information of KEGG pathway analysis is shown in Supplemental File S5.

### 2.5. Gene Expression Validation by RT-PCR

**Figure 8:**
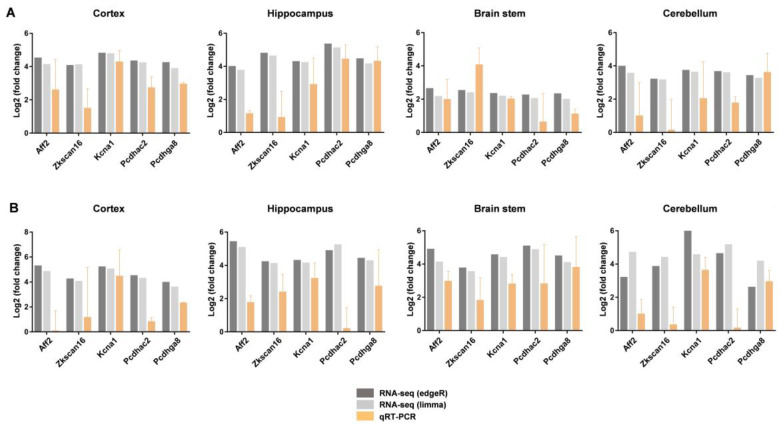
Validation of gene expression by qRT-PCR in DAPK1-KO mice. qRT-PCR analysis of Aff2, Zkscan16, Kcna1, Pcdhac2, and Pcdhga8 in the cerebral cortex, hippocampus, brain stem, and cerebellum for males (**A**) and females (**B**). Each data point represents the mean ± standard deviation (SD) of three mice.

To confirm the expression of DEGs by RNA-seq, we selected five of the most upregulated genes overlapping in four brain regions for both males and females and performed qRT-PCR with three independent samples. AF4/FMR2 family member 2 (Aff2), zinc finger with KRAB and SCAN domains 16 (Zkscan16), potassium voltage-gated channel subfamily A member 1 (Kcna1), protocadherin alpha subfamily C, 2 (Pcdhac2), and protocadherin gamma subfamily A, 8 (Pcdhga8), which are upregulated in the tissues of all four brain regions for both males and females, were selected and analyzed. The RNA-seq data showed that the expression of Aff2, Zkscan16, Kcna1, Pcdhac2, and Pcdhga8 in the cerebral cortex of male DAPK1-KO mice was increased by 2^4.5^ (by edgeR method, 2^4.1^ by limma method), 2^4.0^ (by edgeR method, 2^4.1^ by limma method), 2^4.8^ (by edgeR method, 2^4.8^ by limma method), 2^4.3^ (by edgeR method, 2^4.2^ by limma method), and 2^4.2^ (by edgeR method, 2^3.9^ by limma method) fold changes, compared with that of WT mice, respectively. The data are shown in column ‘logFC’ in Supplemental File S1. Consistently, the results from the qRT-PCR experiment showed that the expression levels of Aff2, Zkscan16, Kcna1, Pcdhac2, and Pcdhga8 were increased by 2^2.6^-, 2^1.5^-, 2^4.2^-, 2^2.7^-, and 2^2.9^-fold, respectively, in the cerebral cortex of male DAPK1-KO mice compared with those of WT mice (Figure 8A). Although the levels of gene expression from qRT-PCR were different compared with those determined in the RNA-seq data, the upregulation pattern was the same in all brain regions for both males and females (Figure 8A,B).

## 3. Discussion

DAPK1 is highly expressed in proliferative and postmitotic regions within the cerebral cortex, hippocampus, and cerebellar Purkinje cells in rats [5]. Moreover, the mRNA level of DAPK1 in the brain increases at the prenatal stages and gradually declines after birth [5]. Interestingly, DAPK1 has been reported to be expressed widely in the brain and it is highly expressed in the cerebral cortex, hippocampus, and cerebellum during the embryonic stages [5]. However, DAPK1 expression was only found to be distributed in the cerebral cortex and hippocampus, not the cerebellum, in the brains of adult rats. This distribution of DAPK1 implies that it might be involved in neuronal functions such as synaptic transmission and plasticity as well as memory and learning [5,42]. To explore the physiological function of DAPK1 in the brain, gene expression profiling was generated and analyzed in the cerebral cortex, hippocampus, brain stem, and cerebellum of DAPK1-KO mice [43]. We found that DAPK1 was expressed in all four brain regions in the adult mouse brain, conflicting with previous reports that indicated DAPK1 was expressed only in the adult rat cerebral cortex and hippocampus [5]. Moreover, the highest number of DEGs was found in the brain stem of male mice, while the highest number of DEGs was found in the cerebellum of female mice. Interestingly, the number of upregulated genes and downregulated genes were similar in the cerebral cortex, brain stem, and cerebellum, whereas the number of upregulated genes was approximately 88% and 75% of the total DEGs in the hippocampus for males and females, respectively. The data suggested that DAPK1 might specifically regulate gene expression in the hippocampus. DEGs are distributed on 20 chromosomes and in the mitochondria but are not restricted to particular chromosomes. Overall, the data indicated that DAPK1 has a wide effect on gene transcription.

GO enrichment analysis showed that transcription related functions, such as regulation of DNA-binding transcription factor activity, DNA-binding transcription repressor activity, transcription coregulator activity, and transcription coactivator activity, were significantly affected by DAPK1 in all brain tissues. This might be the reason why DAPK1 has a global effect on gene expression, even though DAPK1 is not a transcription factor. DAPK1 may regulate the activity and cellular localization of transcription factors through its kinase activity. However, further research is needed to investigate whether and how DAPK1 affects the activity of transcription regulators. Moreover, the cellular component terms involved in ribosomes were enriched in most of the brain tissue regions except the hippocampus. The molecular function terms that involved ribosome and channel activity related functions were enriched. KEGG pathway analysis further supported the results from the GO enrichment analysis. To our surprise, in addition to the ribosome pathway, pathways associated with COVID-19 were most significantly affected by the downregulation of DAPK1. Ribosomal-related proteins have been reported to be widely expressed in the COVID-19 pathway [44]. The data suggest that DAPK1 might play a role in COVID-19. However, the cause of this correlation is not known, and warrants further investigation. Furthermore, we found that the biological process terms of DAPK1-KO brains DEGs were mainly enriched in synaptic, cognitive, and neuron death-associated functions, in which the cerebellum is less relevant to cognition than other brain tissues. KEGG pathway analysis also showed that synapse-related pathways, such as glutamatergic synapses and GABAergic synapses, were significantly enriched in all brain region tissues. In accordance with our data, other studies have reported that DAPK1 has an impact on synaptic and cognitive functions [14,16,23,45,46]. For example, activation of DAPK1 is required for long-term depression (LTD), which is a form of long-term synaptic plasticity that is caused by inhibition of Ca^2+^/calmodulin-dependent protein kinase II (CaMKII) accumulation in the synapse and NMDA-receptor subunit 2B binding (GluN2B) [14]. Another report showed that DAPK1-KO prevents cognitive impairment in vascular cognitive impairment and dementia (VCID) mice [46]. Consistently, DAPK1 has also been demonstrated to regulate neuronal cell death [13,17,25,47,48]. Activation of DAPK1 by extracellular signal-regulated kinase (ERK) increases neuronal apoptosis in an epilepsy mouse model, while DAPK1 gene depletion alleviates neuronal cell death [11]. In addition to ribosome-related pathways, age-associated (including NAFLD pathways, oxidative phosphorylation pathways, as well as many neurodegenerative disease-related pathways) were also the significantly enriched KEGG pathways. Neuronal cell death and cognition were enriched in the GO analysis and corresponded with the KEGG enriched pathways associated with neurodegenerative diseases, verifying the similarity of these two analyses. Consistent with these findings, DAPK1 has been shown to be involved in many neurological disorders, such as AD, PD, and HD. For example, downregulation of DAPK1 attenuated the neuropathology of AD, including Aβ secretion and tau hyperphosphorylation at AD-related sites [9,19,20,29,30].

qRT-PCR analysis confirmed the expression of five genes and indicated that the regulation patterns of most genes followed the same trends as those in the RNA-seq results. Moreover, we found that the fold change of Kcna1 expression was the highest among the five genes. A number of studies have shown that Kcna1 is implicated in epilepsy [48,49,50,51,52,53]. Simeone et al. found that Kcna1-KO mice developed methacholine (MCh)-induced seizures and had greater respiratory sensitivity to MCh when they approached epileptic sudden death. Moreover, Kcna1-KO mice exhibited increased respiratory drive and decreased blood oxygen saturation with a higher probability of epileptic sudden death [48]. These data suggest that the progression of respiratory dysfunction with age may result in higher susceptibility to respiratory failure during severe seizures, consequently increasing the risk of epileptic sudden death in Kcna1-KO mice [48]. In addition, the potassium channel genes KCNA1, KCNA2, KCNB1, KCNC1, KCND2, KCNQ2, KCNQ3, KCNMA1, and KCNT1 have been implicated in epilepsy [52]. Consistently, our data revealed that potassium channel-related GO terms, such as potassium ion transmembrane transport, potassium ion transport, potassium channel complex, potassium channel activity, and voltage-gated potassium channel complex, were significantly enriched in the DEGs of DAPK1-KO mice. These findings implied that DAPK1 might be strongly involved in epilepsy. Our previous studies have validated that DAPK1 plays a critical role in the development of epilepsy and might be a potential target for neuronal protection in epilepsy [10,11]. However, whether inhibition of DAPK1 protects against epileptic seizures through potassium channels deserves further study.

## 4. Materials and Methods

### 4.1. Animals

WT C57BL/6 mice were obtained from Shanghai Laboratory Animal Research Center (Shanghai, China) and the generation of DAPK1-KO mice on the C57BL/6 background was described previously [43]. All mice were maintained on a 12-h light/dark cycle with water and food supply in the SPF facility of Fujian Medical University. 

### 4.2. Immunoblotting Analysis

The tissues from the cerebral cortex, hippocampus, brain stem, and cerebellum were lysed using radioimmunoprecipitation assay buffer (RIPA buffer) containing protease and phosphatase inhibitor cocktails (Transgene, Beijing, China). The protein concentration was measured using a BCA protein assay kit (Beyotime, Shanghai, China). Then, the protein was incubated with loading buffer at 95 °C for 10 min. Protein samples (5–10 μg) were separated by 10% SDS-PAGE and transferred to 0.45-μm polyvinylidene fluoride membranes (MilliporeSigma, St.Louis, MO, USA), followed by blocking with 5% milk-TBST at room temperature for 1 h. The membranes were incubated with an anti-DAPK1 antibody (MilliporeSigma, Cat# D2178) or an anti-β-actin antibody (MilliporeSigma, Cat# A5441) at 4 °C overnight, and then with an HRP-conjugated secondary antibody at room temperature for 1 h after washing in TBST. The membranes were further exposed using enhanced chemiluminescence HRP substrate (MilliporeSigma) on the Bio-Rad Chemidoc imaging system (Bio-Rad, Hercules, CA, USA). 

### 4.3. Transcriptome Sequencing

The tissues from each brain region were dissected from 6 WT (3 male and 3 female) and 6 DAPK1-KO (3 male and 3 female) mice at the age of 12 months. Total RNA was extracted from mouse brain tissues using TRIzol and assessed by the RNA Nano 6000 Assay Kit and the Agilent Bioanalyzer 2100 system (Agilent Technologies, Santa Clara, CA, USA). The minimum RNA integrity number (RIN) for the samples is four. The construction of sequencing library was performed using the NEBNext Ultra RNA Library Prep Kit (NEB, Ipswich, MA, USA), followed by sequencing on the Illumina NovaSeq 6000 system (Novogene Corp., Sacramento, CA, USA). 

### 4.4. Bioinformatic Analysis

DEGs between the two groups were first identified using the R software ‘limma’ package (v3.28.14) and ‘edgeR’ package (v3.14.0), respectively [54,55]. The Benjamin–Hochberg method was applied to control the false discovery rate (FDR) [56]. Genes with |log_2_FC| > 1 and FDR < 0.05 were considered DEGs regulated by DAPK1 and were further visualized by the R software package ggplot2 (v3.3.3) [57]. Subsequently, identifying DEGs by overlapping these two methods increased the reliability of statistical analyses. In addition, we also conducted enrichment analyses to explore whether these DEGs were significantly enriched in predefined biological KEGG pathways or GO terms by using the ‘clusterProfiler’ R package (v4.0) [58]. The KEGG database represents the most comprehensive collection of manually drawn pathway maps of molecular interactions and is utilized as a reference to map newly identified DEGs [59]. The GO terms describe the biological domain of a gene with respect to three categories: molecular function, cellular component, and biological process [60].

### 4.5. Quantitative RT-PCR Assay

Real-time qRT-PCR was conducted using the QuantStudio Real-Time PCR System (Thermo Fisher Scientific, Waltham, MA, USA) as described previously [61]. The primers used in this study were as follows: Aff2, forward 5′- CTTGGAGCAGCAGTGTCACTAT-3′, reverse 5′- AGGGCATCCCCTTTGTTTGTAT-3′; Zkscan16, forward 5′-GTTGAACAGCGTCTCTGGCT-3′, reverse 5′-CAGTCTTGAAGGAACTGGGACT-3′; Kcna1, forward 5′-GGGTAGGGTACGGACGTTTC-3′, reverse 5′-GATCGATGGACGCTGGC-3′; Pcdhac2, forward 5′-CTGGCAGTCGCAGAAAATCG-3′; reverse 5′-ACTACAAATGCCCGAGACGG-3′; Pcdhga8, forward 5′-AGGATGAAGATGCTTGCGCT-3′, reverse 5′-TCACCATTTTGGGATCCGCT-3′; β-actin, forward 5′-GTGACGTTGACATCCGTAAAGA-3′, reverse 5′-GCCGGACTCATCGTACTCC-3′. The tissues from each brain region were dissected from six WT (three male and three female) and six DAPK1-KO (three male and three female) mice. Each sample was amplified in duplicate. Data were analyzed by the comparative Ct (ΔΔCt) method by normalizing expression to β-actin.

## 5. Conclusions

In summary, we analyzed global gene expression changes in the brains of DAPK1-KO mice compared with that of WT mice for the first time. Transcriptional profiling showed that more genes were upregulated than downregulated in all brain regions, especially in the hippocampus. We also found that synaptic, cognitive, and neuronal death-associated functions were most dramatically enriched in the biological process category in the GO enrichment analysis. Moreover, in addition to ribosome-related and COVID-19 pathways, neurodegeneration-related pathways were significantly enriched in the brains of DAPK1-KO mice. We also found that Kcna1, which has been implicated to be involved in epilepsy, was highly upregulated in DAPK1-KO mice. In conclusion, our study revealed an effect of DAPK1 on neuronal functions and might provide insights into the development of novel therapeutic strategies for neurological diseases.

## Figures and Tables

**Table 1:** GO categories of DEGs.

Gender	Tissues	Total GO Categories	Biological Processes	Cell Component	Molecular Function
Male	Cortex	983	727	139	117
	Hippocampus	79	29	26	24
	Brain stem	930	691	135	104
	Cerebellum	440	275	107	58
Female	Cortex	244	156	61	27
	Hippocampus	32	21	1	10
	Brain stem	271	160	70	41
	Cerebellum	267	129	94	44

## Data Availability

All data generated or analyzed during this study are available from the corresponding author on reasonable request.

## Supplementary Materials

The following supporting information can be downloaded at: https://www.mdpi.com/article/10.3390/ijms24076542/s1.
