# Supplementary material for: Ablation of Death-Associated Protein Kinase 1 Changes the Transcriptomic Profile and Alters Neural-Related Pathways in the Brain
Source: Int J Mol Sci. 2023 Mar 31;24(7):6542. doi: 10.3390/ijms24076542 (PMC10095516; doi:10.3390/ijms24076542)
Supplement: Supplementary file 1 [file ijms-24-06542-s001.zip › ijms-2279477-supplementary/Supplemental File 6.pdf]

Supplementary Data

Li et al.

Ablation of Death-associated Protein Kinase 1 Changes the Transcriptomic Profile and Alters Neural-related Pathways in the Brain

Supplemental figure legends

Figure S1. Transcriptional profiling of different brain region tissues in DAPK1-KO mice. (A) Differential expression volcano plots for tissues in each brain region for male mice by the limma method. The horizontal dotted line refers to the threshold of statistical significance with log, while the vertical dotted line refers to the threshold of the differential expressed ratio. (B) Number of DEGs in four brain region tissues for female mice.

Figure S2. Differential expression volcano plots for tissues in each brain region in DAPK1-KO female mice by the edgeR (A) and limma (B) methods. The horizontal dotted line refers to the threshold of statistical significance with log, while the vertical dotted line refers to the threshold of the differential expressed ratio.

Figure S3. Chromosome distribution of significantly regulated genes in different brain region tissues of female DAPK1-KO mouse brains. Chromosome distribution of DEGs in the cerebral cortex (A), hippocampus (B), brain stem (C) and cerebellum (D). (E) Chromosome distribution of the common DEGs in all four brain regions.

Figure S4. Chromosome distribution of the common DEGs in all four brain regions in male (A) and female (B) DAPK1-KO mice.

Figure S5. Gene ontology enrichment analysis of the DEGs in the cerebral cortex (A) and hippocampus (B) of female DAPK1-KO mice. The GO categories were: biological process (BP), cellular component (CC), and molecular function (MF).

Figure S6. Gene ontology enrichment analysis of the DEGs in the brain stem (A) and cerebellum (B) of female DAPK1-KO mice. The GO categories were: biological process (BP), cellular component (CC), and molecular function (MF).

Figure S7. KEGG pathway analysis of the DEGs in the cerebral cortex (A) and hippocampus (B) of female DAPK1-KO mice.

Figure S8. KEGG pathway analysis of the DEGs in the brain stem (A) and cerebellum (B) of female DAPK1-KO mice.

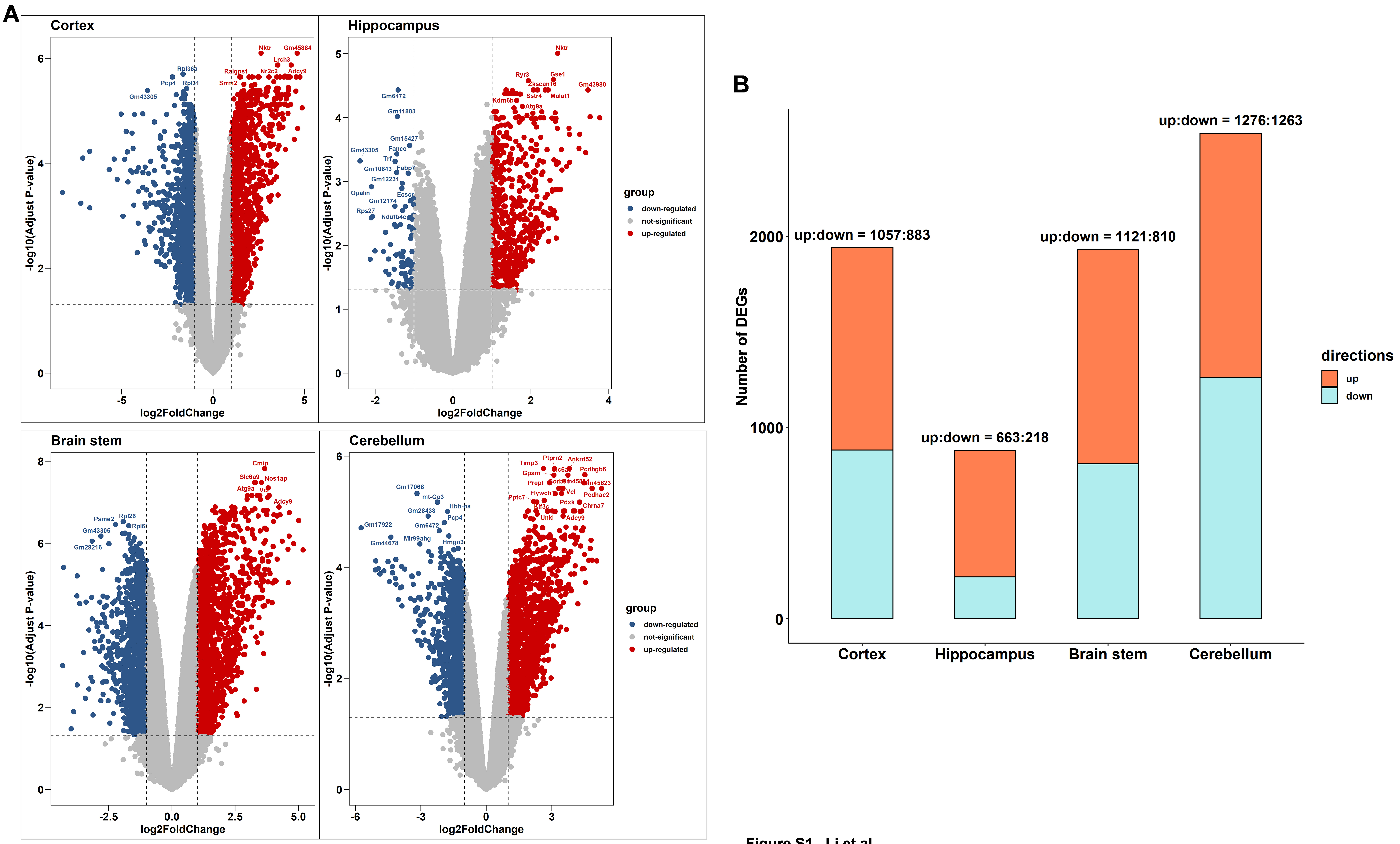

Figure S1. Li et al.

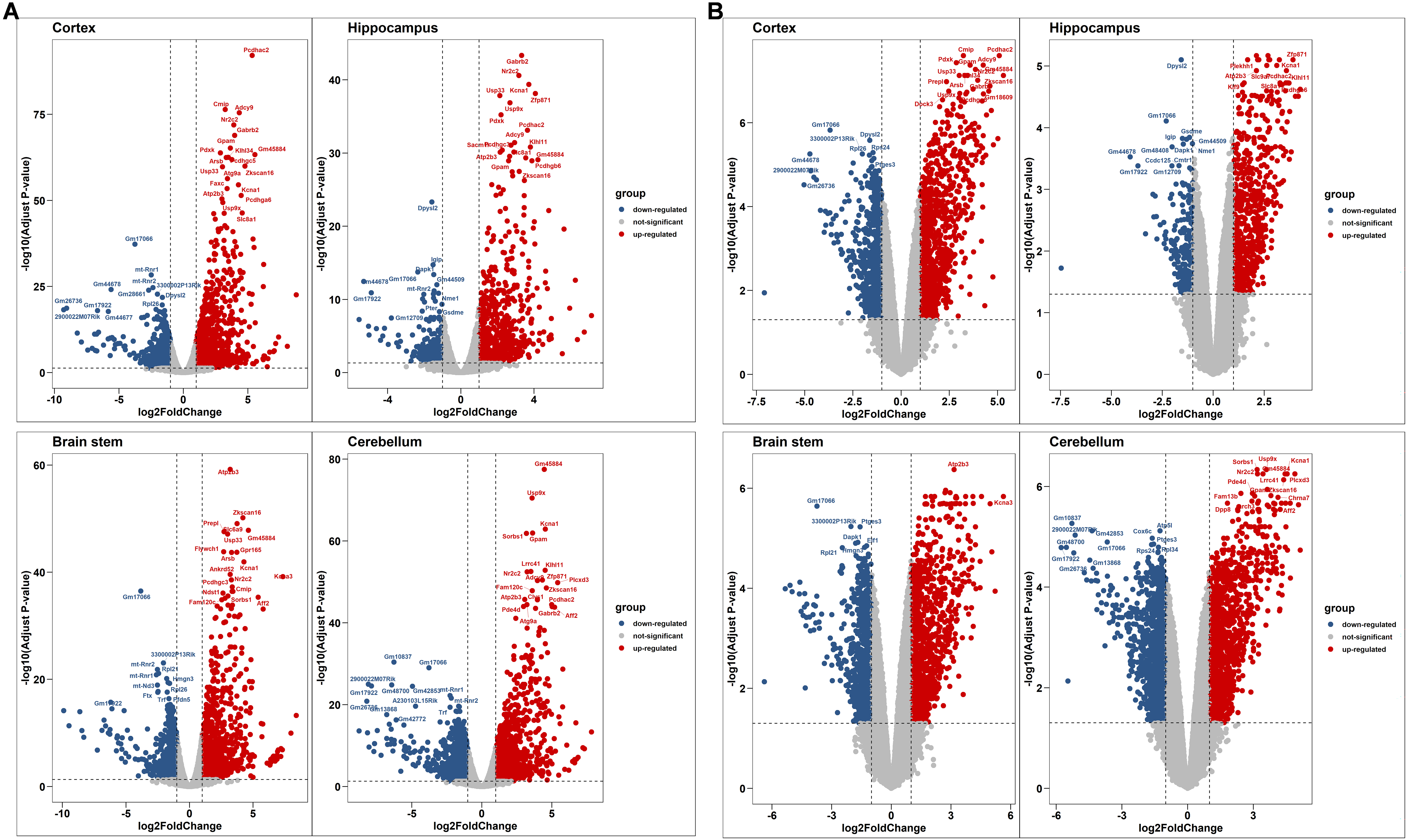

Figure S2. Li et al.

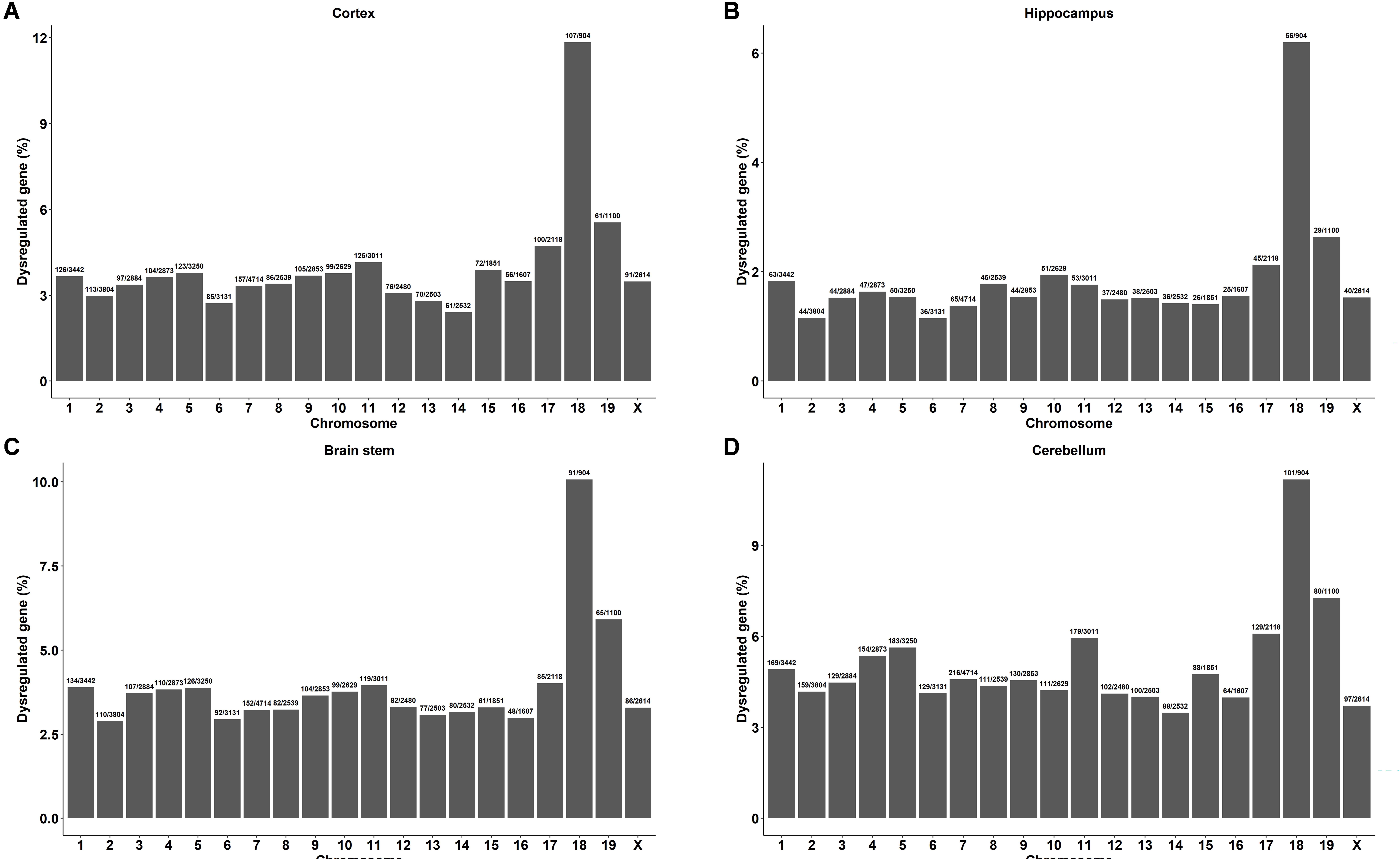

Figure S3. Li et al.

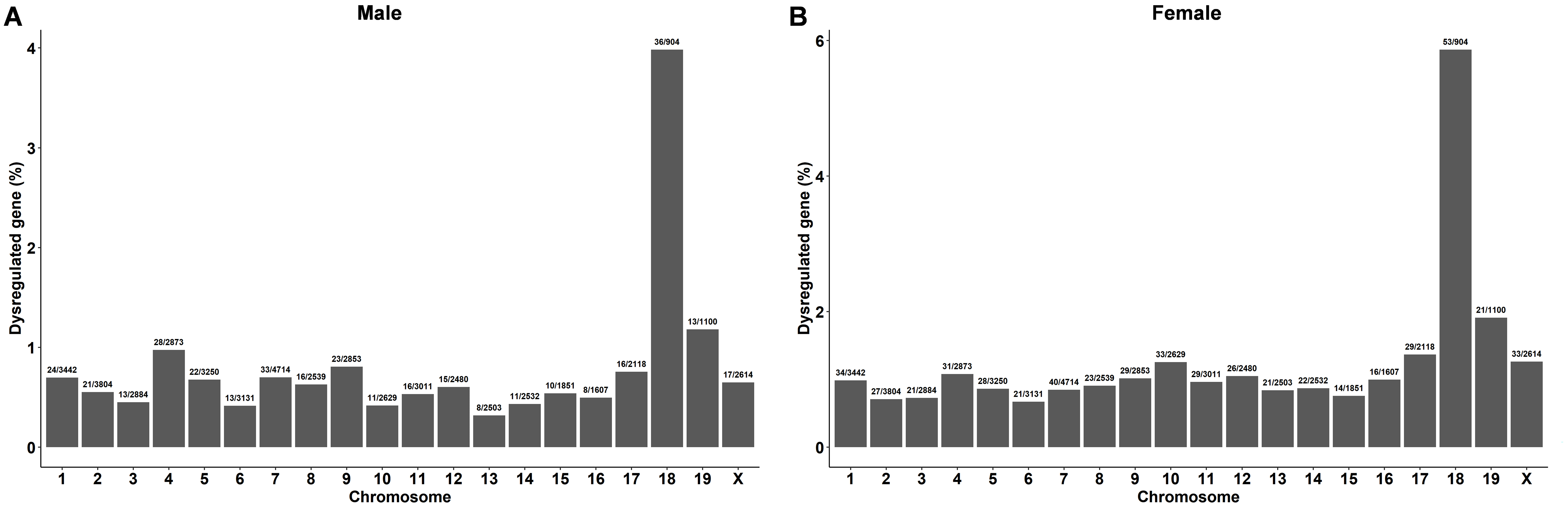

Figure S4. Li et al.

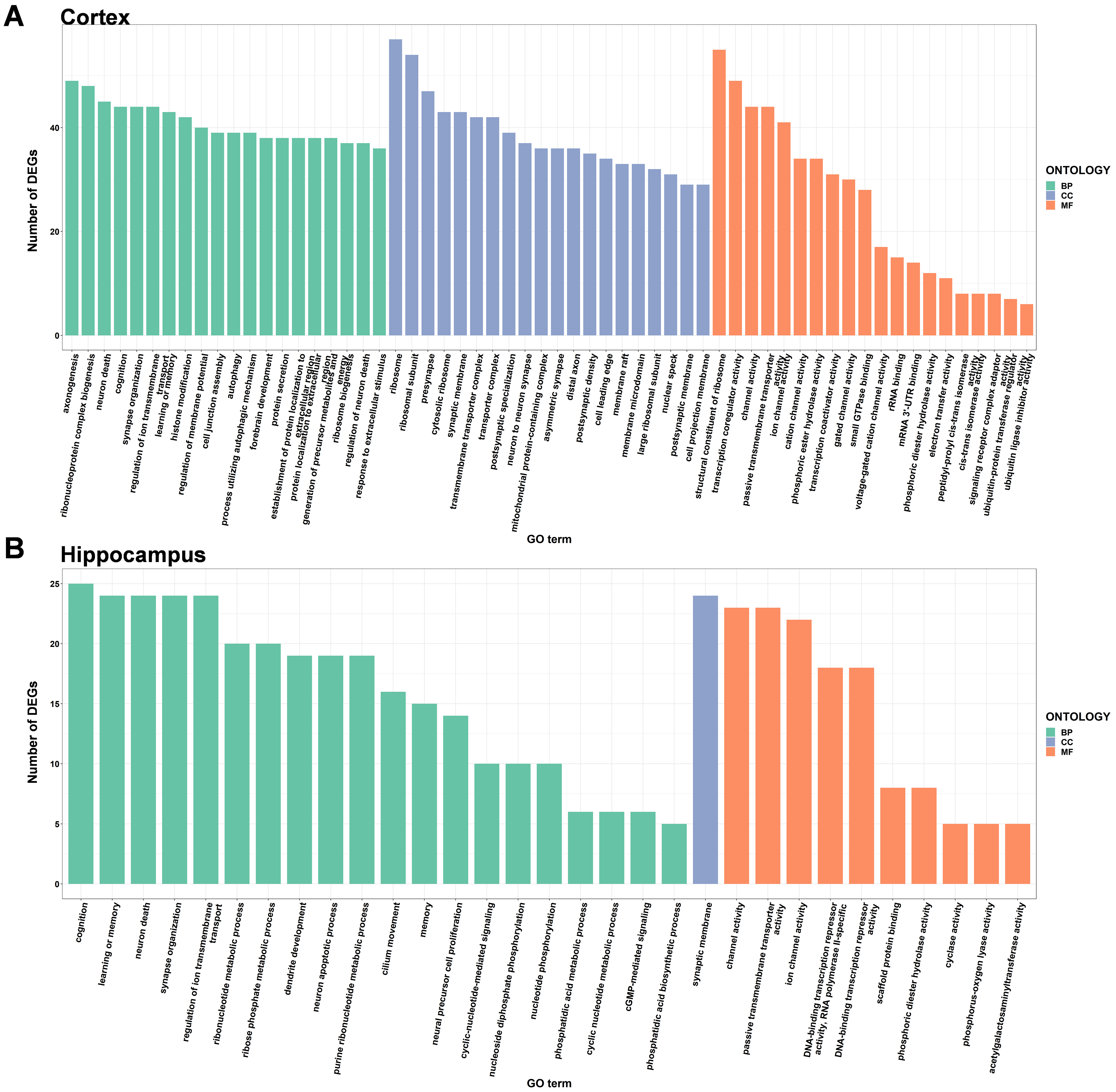

Figure S5. Li et al.

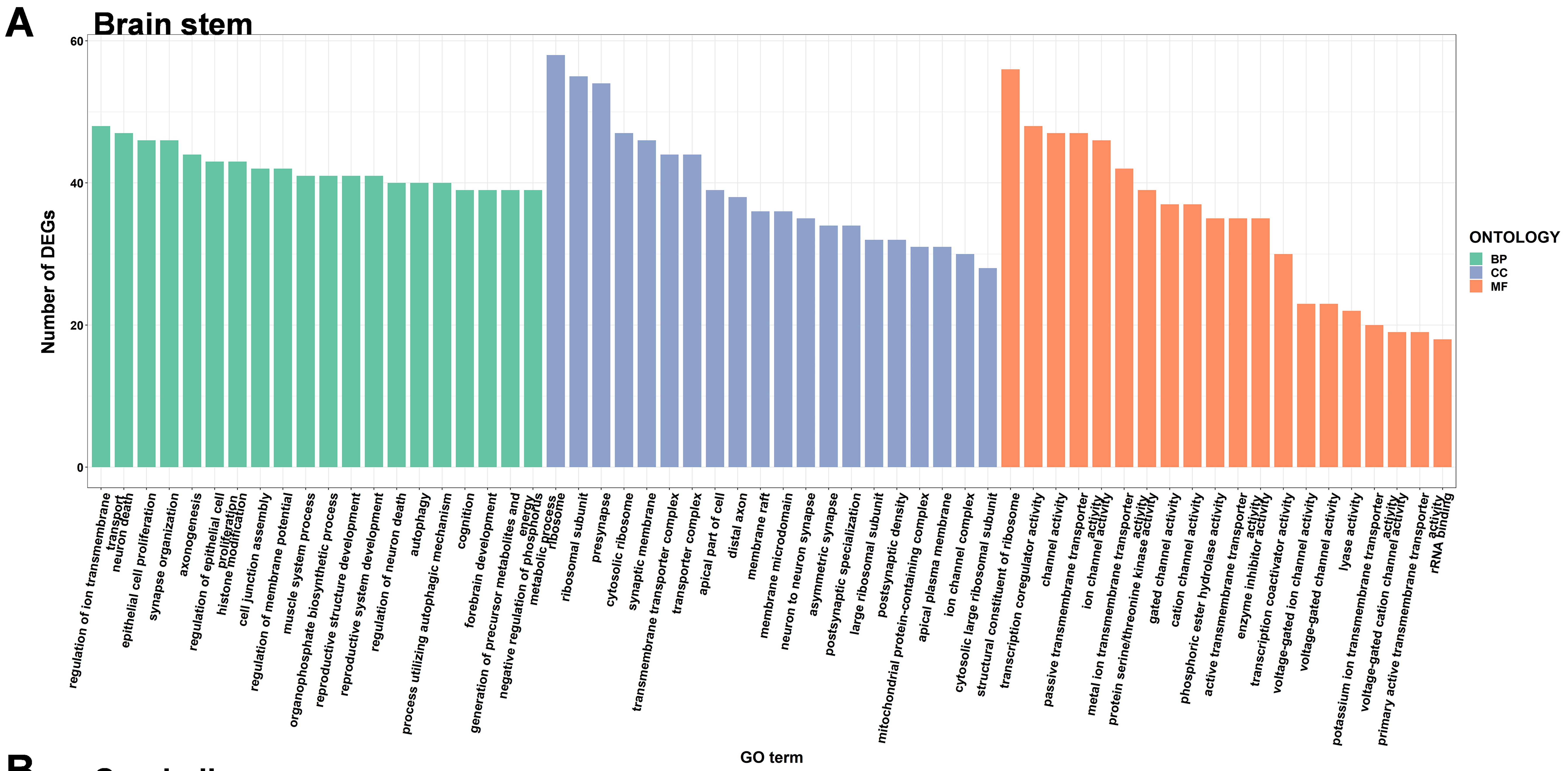

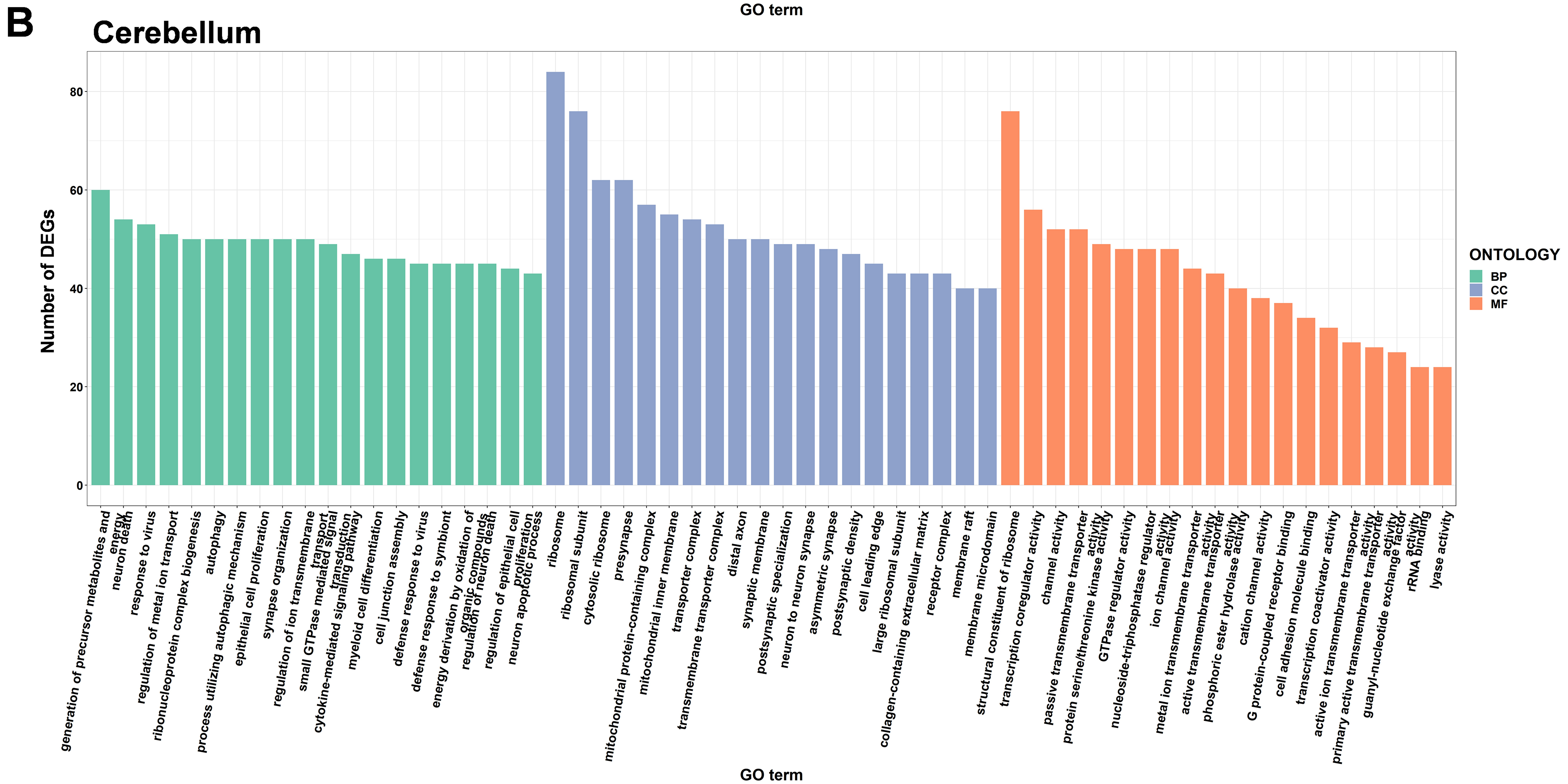

Figure S6. Li et al.

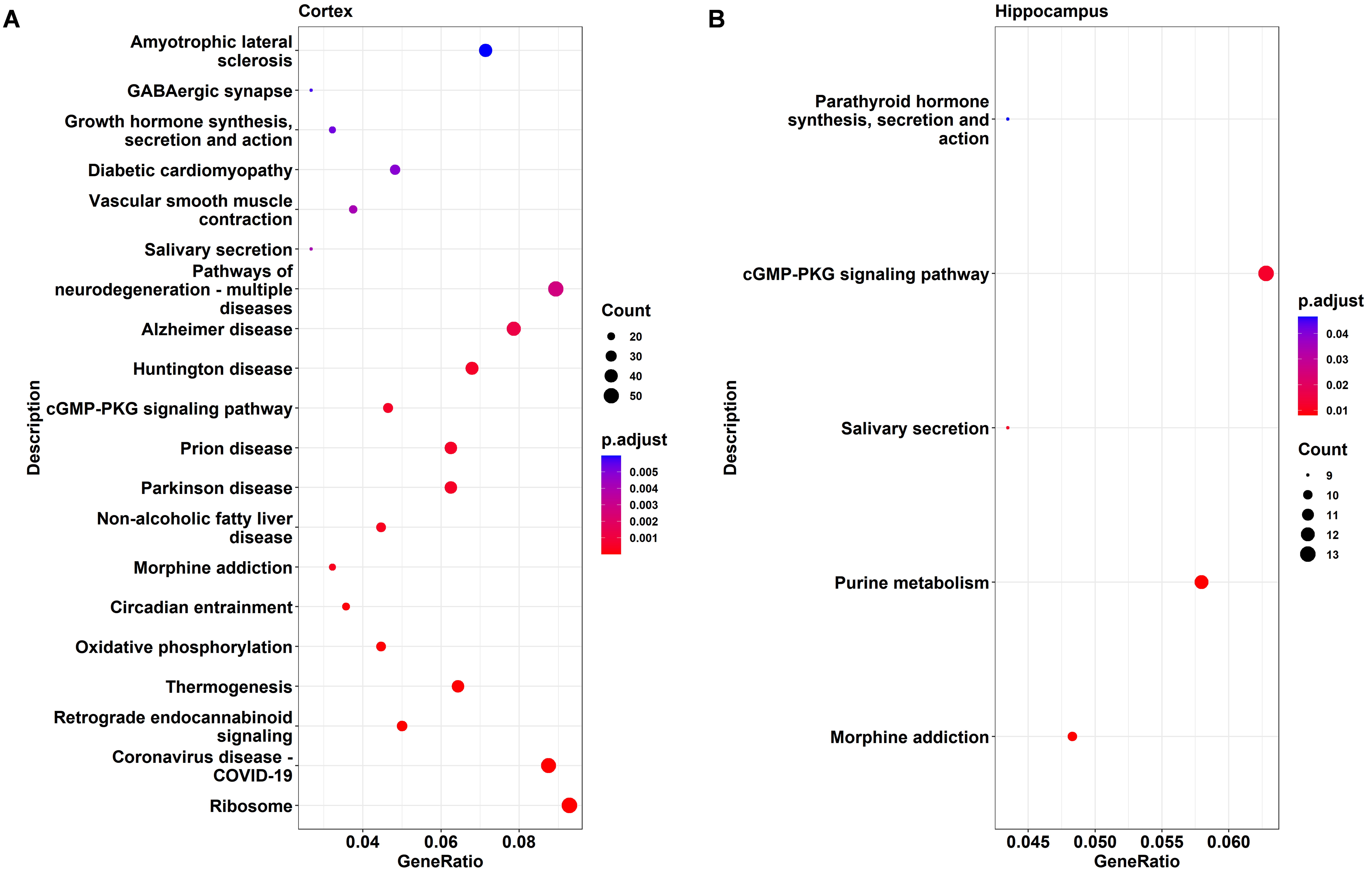

Figure S7. Li et al.

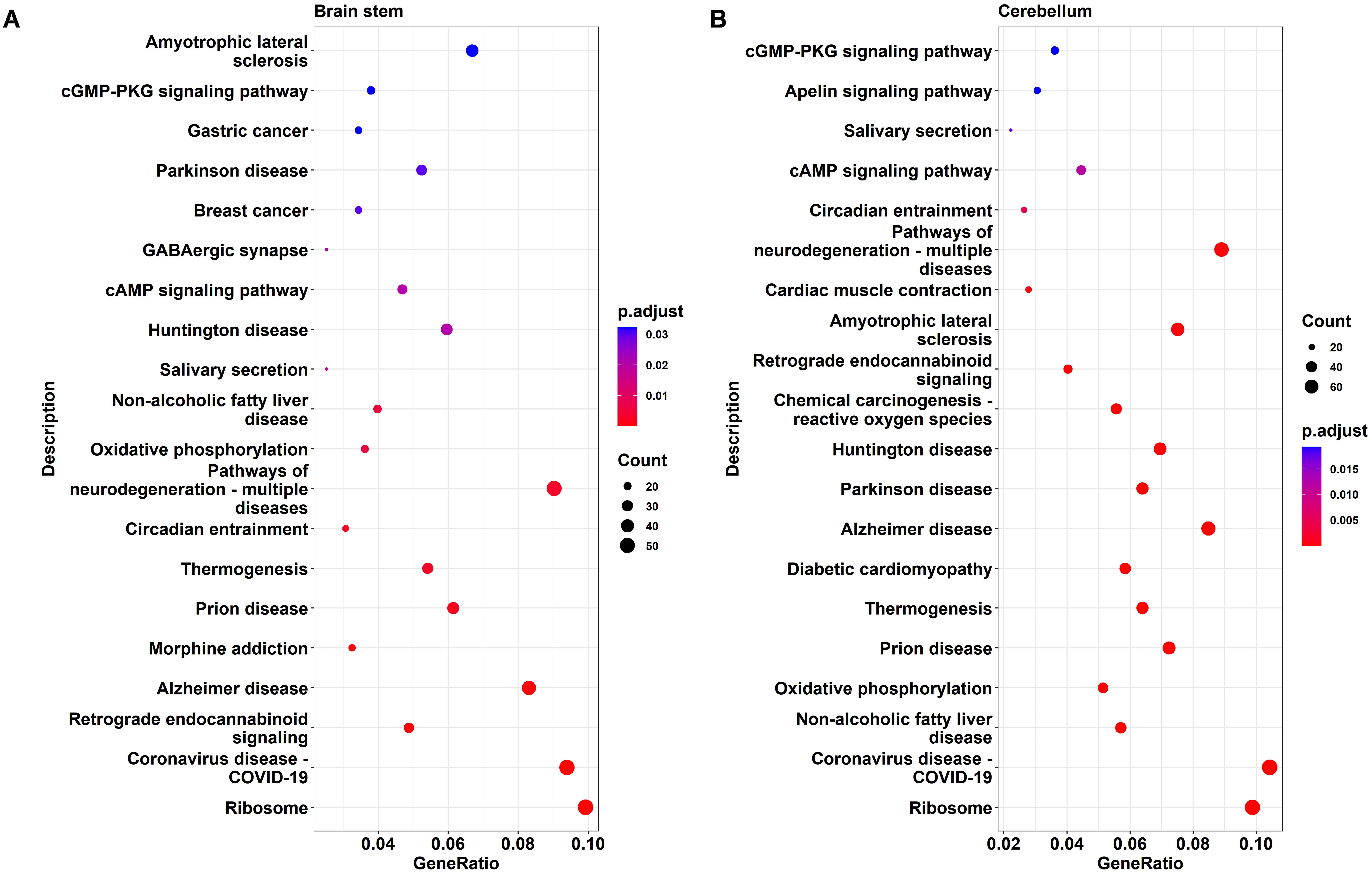

Figure S8. Li et al.
